# Cerebral monitoring in a pig model of cardiac arrest with 48 h of intensive care

**DOI:** 10.1186/s40635-022-00475-2

**Published:** 2022-10-26

**Authors:** Lauge Vammen, Cecilie Munch Johannsen, Andreas Magnussen, Amalie Povlsen, Søren Riis Petersen, Arezo Azizi, Michael Pedersen, Anders Rosendal Korshøj, Steffen Ringgaard, Bo Løfgren, Lars W. Andersen, Asger Granfeldt

**Affiliations:** 1 Department of Anesthesiology and Intensive Care, Aarhus University Hospital, Palle Juul Jensens Blvd. 99 G304, 8200 Aarhus N, Denmark; 2 Department of Clinical Medicine, Aarhus University, Aarhus N, Denmark; 3 Department of Cardiothoracic Anesthesia, Copenhagen University Hospital, Rigshospitalet, Copenhagen, Denmark; 4 Comparative Medicine Laboratory, Aarhus University, Aarhus N, Denmark; 5 Department of Neurosurgery, Aarhus University Hospital, Aarhus N, Denmark; 6 MR Research Centre, Aarhus University, Aarhus N, Denmark; 7 Research Center for Emergency Medicine, Aarhus University Hospital, Aarhus N, Denmark; 8 Department of Medicine, Randers Regional Hospital, Randers, Denmark; 9 Prehospital Emergency Medical Services, Central Denmark Region, Aarhus N, Denmark

**Keywords:** Heart arrest, Acute myocardial infarction, Post-cardiac arrest intensive care, Targeted temperature management

## Abstract

**Background:**

Neurological injury is the primary cause of death after out-of-hospital cardiac arrest. There is a lack of studies investigating cerebral injury beyond the immediate post-resuscitation phase in a controlled cardiac arrest experimental setting.

**Methods:**

The aim of this study was to investigate temporal changes in measures of cerebral injury and metabolism in a cardiac arrest pig model with clinically relevant post-cardiac arrest intensive care. A cardiac arrest group (*n* = 11) underwent 7 min of no-flow and was compared with a sham group (*n* = 6). Pigs underwent intensive care with 24 h of hypothermia at 33 °C. Blood markers of cerebral injury, cerebral microdialysis, and intracranial pressure (ICP) were measured. After 48 h, pigs underwent a cerebral MRI scan. Data are presented as median [25th; 75th percentiles].

**Results:**

Return of spontaneous circulation was achieved in 7/11 pigs. Time to ROSC was 4.4 min [4.2; 10.9]. Both NSE and NfL increased over time (*p* < 0.001), and were higher in the cardiac arrest group at 48 h (NSE 4.2 µg/L [2.4; 6.1] vs 0.9 [0.7; 0.9], *p* < 0.001; NfL 63 ng/L [35; 232] vs 29 [21; 34], *p* = 0.02). There was no difference in ICP at 48 h (17 mmHg [14; 24] vs 18 [13; 20], *p* = 0.44). The cerebral lactate/pyruvate ratio had secondary surges in 3/7 cardiac arrest pigs after successful resuscitation. Apparent diffusion coefficient was lower in the cardiac arrest group in white matter cortex (689 × 10^–6^ mm^2^/s [524; 765] vs 800 [799; 815], *p* = 0.04) and hippocampus (854 [834; 910] vs 1049 [964; 1180], *p* = 0.03). *N*-Acetylaspartate was lower on MR spectroscopy in the cardiac arrest group (− 17.2 log [− 17.4; − 17.0] vs − 16.9 [− 16.9; − 16.9], *p* = 0.03).

**Conclusions:**

We have developed a clinically relevant cardiac arrest pig model that displays cerebral injury as marked by NSE and NfL elevations, signs of cerebral oedema, and reduced neuron viability. Overall, the burden of elevated ICP was low in the cardiac arrest group. A subset of pigs undergoing cardiac arrest had persisting metabolic disturbances after successful resuscitation.

**Supplementary Information:**

The online version contains supplementary material available at 10.1186/s40635-022-00475-2.

## Introduction

The number of published studies including a clinically relevant cardiac arrest animal model is limited [1]. To address this, we have developed a cardiac arrest pig model with myocardial infarction and 48 h of post-resuscitation care [2]. In that model, we found significantly depressed cardiovascular function as well as signs of renal, hepatic, and pulmonary injury during the post-cardiac arrest phase.

Neurological injury is the primary cause of death after out-of-hospital cardiac arrest [3]. A multimodal approach to neuroprognostication, and non-invasive cerebral injury assessment, is preferred in the clinical setting [4]. This often includes magnetic resonance imaging (MRI) and biomarkers of neurological injury [5]. Few studies on post-cardiac arrest patients have used intracranial pressure (ICP), brain oxygenation (PTiO_2_), and microdialysis to assess cerebral injury [6–9]. However, invasive cerebral measurements are not common in clinical practice due to risk of bleeding and infection. Several cardiac arrest pig studies have utilized ICP, PTiO_2_, and microdialysis monitoring [10–15]. Common to most of these studies are measurements only being collected during the intra-arrest and early return of spontaneous circulation (ROSC) periods, with only two investigations having an observation period beyond 6 h [16, 17]. A multimodal description of long-term cerebral injury development in a controlled experimental setting is therefore warranted.

The aim of this study was to investigate temporal changes in measures of cerebral injury and metabolism in a cardiac arrest pig model with clinically relevant post-cardiac arrest intensive care.

## Methods and materials

The study was approved by the Danish Animal Experiments Inspectorate (License number: 2019-15-0201-01647) and conducted and reported in accordance with the ARRIVE [18] and Utstein-Style [19] guidelines. This manuscript describes cerebral outcome data from a previously published experiment. Detailed methodological descriptions are described therein [2]. Animals were assigned to the groups at the discretion of the primary investigator in a non-randomized order. The investigators were not blinded to group allocation.

### Animals

Female crossbred Landrace/Yorkshire/Duroc pigs (40 kg) were fasted overnight with free access to water.

### Animal preparation

Esketamine (6.25 mg/kg), midazolam (0.625 mg/kg), and atropine (0.5 mg) were used to induce anaesthesia, which was maintained with propofol (4.0–5.5 mg/kg/h) and remifentanil (0.6–1.0 µg/kg/min). Pigs were intubated and ventilated at a tidal volume of 8 ml/kg with pressure-controlled volume guarantee settings (Datex Ohmeda S5, GE Healthcare, IL, USA). The ventilator at baseline was adjusted as follows: respiratory rate to control arterial partial CO_2_ pressure (PaCO_2_) between 4.7 and 6.0 kPa, fraction of inspired O_2_ (FiO_2_) to maintain arterial partial O_2_ pressure (PaO_2_) between 20–25 kPa, and positive end-expiratory pressure (PEEP) at 5 cmH_2_O. Temperature was regulated at baseline to normothermia (38.5 ± 0.5 °C).

### Invasive cerebral monitoring

The pig was placed in prone position and two burr holes were made in the skull 1.5 cm lateral to the sagittal suture and 1.5 cm caudal to the coronal suture. Both burr holes were fitted with a bolt (Bolt Kit, Raumedic AG, Helmbrechts, Germany). The pig was turned back to supine position and after puncture of the dura mater a catheter for measurement of intracranial pressure (ICP), brain tissue oxygenation (PTiO_2_), and temperature (NEUROVENT-PTO, Raumedic AG, Helmbrechts, Germany) was inserted in the right parietal lobe parenchyma and connected to a monitor (MPR2 logO Datalogger, Raumedic AG, Helmbrechts, Germany). Cerebral perfusion (CerPP) was calculated as MAP-ICP. ICP and PTiO2 data are reported as timepoint values. In the bolt over the left parietal lobe, a customized microdialysis catheter (CMA 20 Elite 10 mm, 20 kD cut-off, CMA CFM, Marseille, France) was inserted into the brain parenchyma. The microdialysis catheter was perfused with an artificial CNS fluid (Perfusion fluid CNS, CMA CFM) at a flow rate of 0.5 µL/min (CMA 402 Microdialysis Syringe Pump, CMA CFM). The dialysate samples were collected in microvials and stored at − 80 °C for later analysis (CMA 600 Microdialysis Analyzer, CMA CFM). The pre-cardiac arrest baseline sample was collected after 60 min of tissue recovery time for a sampling period of 60 min. After ROSC samples were collected every two hours. No dialysate samples were collected during cardiac arrest (sampling vials were exchanged 5 min after ROSC to collect the first post-resuscitation sample). The following metabolic markers were analysed: lactate, pyruvate, glucose, and glycerol (Reagent Set A, µdialysis, Stockholm, Sweden). Recovery rates of analytes in the dialysate were not calculated.

### Experimental protocol

Induction of myocardial infarction, cardiac arrest, resuscitative efforts, and the post-cardiac arrest intensive care protocol for this experiment have been described in detail previously [2]. The earlier report included two different methods of myocardial infarction. One group had a 45 min continuous coronary occlusion starting 10 min prior to cardiac arrest induction and lasting throughout cardiac arrest, resuscitation, and the early post-ROSC phase. This resulted in severe cardiovascular instability during the post-cardiac arrest period (AMI-Cont group). The other method included an intermittent coronary reperfusion during cardiac arrest and resuscitation (see details below), which resulted in moderate cardiovascular instability during the post-cardiac arrest period (AMI-Int). This manuscript only reports neurological data from the AMI-Int group, as the AMI-Cont group only consisted of two animals surviving the entire protocol. Data from the AMI-Cont group are, however, presented in the supplemental material. In the main manuscript, the AMI-Int group is referred to as the cardiac arrest group.

In short, myocardial infarction was induced by endovascular balloon occlusion of the left anterior descending artery after the second diagonal branch. After 10 min of occlusion, the coronary artery was reperfused for 2 min, and then ventricular fibrillation (VF) was induced by a 9-V DC current delivered to the right ventricle. After a no-flow period of 7 min, mechanical chest compressions (LUCAS2) and ventilations were initiated. Five minutes after ROSC, the balloon catheter was re-inflated for another 35 min of coronary occlusion. Furthermore, goal-directed intensive care was initiated with special emphasis on cardiovascular stability after set treatment goals (for details see Additional file 1: Methods and Table S1). One hour after ROSC, targeted temperature management (TTM) was initiated to cool the pigs to 33 °C and maintain this temperature for 24 h before rewarming to normothermia (38.5 °C ± 0.5) by 0.5 °C per hour. For comparison, a group of sham animals was included and underwent the same procedures except for myocardial infarction, cardiac arrest, and subsequent resuscitative efforts.

#### Magnetic resonance imaging

After 48 h of intensive care the pigs were transported to an MRI scanner (Achieva DStream 1.5 T, Phillips, Best, The Netherlands). For details see *Supplemental Methods*. The perfusion scans were analysed by a semi-quantitative method, creating parametric maps of the upslope of the signal intensity–time curve (MIStar, Apollo, Melbourne, Australia). For the calculated apparent diffusion coefficient (ADC), blood oxygenation level dependent (BOLD) and relative perfusion maps, regions of interest (ROIs) included frontal cortex white matter and grey matter, thalamus, hippocampus, and cerebellum. Each cortical ROI were ~ 0.05 cm^2^ while non-cortical ROIs were ~ 0.1 cm^2^. An online histological/MRI atlas of the Göttingen minipig brain was used to guide ROI placement [20]. For the analysis of MRI scans Horos software version 3.3.5 (Annapolis, MD, USA) was used. All ROI placements were checked by an experienced neurosurgeon blinded to group allocation before analysis. All ROIs were drawn by free hand to best fit the anatomical area of interest (see Additional file 1: Fig. S1). For proton-spectroscopy a single whole brain voxel was applied.

#### Blood sampling and analysis

Serum samples were collected at baseline and throughout the post-resuscitation phase to measure neuron-specific enolase (NSE) and neurofilament light chain (NfL). After clotting for a minimum of 20 min, serum samples were centrifuged (1850 G, 4 °C, 9 min) and supernatants stored at − 80 °C for later analysis. NSE was analysed by an electrochemiluminescent immunoassay using Cobas 8000 e602 (Roche Diagnostics GmbH, Mannheim, Germany; analytical variation 11.3 µg/L: ± 10%). NfL was measured by the NF-light^®^ assay using the ultrasensitive SimoaTM HD-1 platform (Quanterix^©^, Lexington, MA, USA; analytical variation 12.3 ng/L: ± 20%).

### Statistics

Data in figures are presented as median with corresponding 25% and 75% quartiles. ICP, MAP, PaO_2_, and NSE from the same experiments have previously been reported [2], but to sufficiently interpret the dataset of this manuscript these variables have been included. Statistical significance was defined as a two-sided *p*-value < 0.05. Study data were collected using REDCap electronic data capture tools hosted at Aarhus University [21]. Data analysis and figures were produced in Stata 17.0 (StataCorp, TX, USA). No sample size calculation was performed due to the descriptive nature of the study.

#### Invasive cerebral, cardiopulmonary, and metabolic data

Data are presented and interpreted using descriptive statistics and a between-group test at baseline and 48 h after resuscitation. If data were approximately normally distributed and had equal variance, an unpaired *t*-test was applied. If data were right-skewed, it was log transformed. Normality was evaluated by qq-plots and equal variance tested by Bartlett’s test. If assumptions for parametric tests were not met a Mann–Whitney *U* test was applied. A Spearman correlation analysis was conducted between PTiO_2_ and PaO_2_. Microdialysis data were only analysed descriptively because of the numerous repeated measurements and no statistical tests were applied.

#### Blood samples

NSE and NfL were analysed with a linear mixed effects model with time (as a categorical value), group and their interaction as fixed effects and individual animals as random effects. Residual errors were structured from an exponential autocorrelation function to allow for unequal spaced categorical time. For NSE, variance differed between the two groups, and this was incorporated into the model. The model was used for checking differences in development over time in the two groups and between-group differences at baseline and 48 h post-resuscitation. Both NSE and NfL were analysed on log-scale, but group differences are presented on normal scale as ratios of geometric means with 95% confidence intervals. The models were checked by qq-plots of residuals and scatter plots of residuals vs. fitted values. Finally, a Spearman correlation analysis was carried out between NSE and NfL measurements from the cardiac arrest group.

#### MR data

Statistical differences between groups in ADC, BOLD sequence, and perfusion maps were tested by unpaired t-test or Mann–Whitney *U* test for each anatomical location. Proton spectroscopy data were analysed with LCModel (Stephen Provencher, Oakville, ON, Canada) version 6.3–1 M. Concentrations of lactate, *N*-acetylaspartate (NAA), choline, and glutamate/glutamine are presented in arbitrary units on log10 scale.

## Results

As previously reported, 17 pigs were included in this study and divided in the two groups (11 cardiac arrest and 6 sham pigs) [2]. Seven pigs in the cardiac arrest group achieved ROSC (baseline data from all pigs are included in analyses). Time to ROSC was 4.4 min [4.2; 10.9]. One pig from the cardiac arrest group died from a tension pneumothorax at 37 h post-resuscitation, another pig from the cardiac arrest group died from fatal arrhythmia in the MRI scanner (only missing perfusion-weighted scan), and one pig from the sham group had an accidental bleeding from arterial catheter after 5 h. Data from all three animals are included up until time of death. In the AMI Cont cardiac arrest group, seven pigs were included. Three achieved ROSC, but one died just before MRI scan due to cardiovascular failure. Data from this group are presented in supplemental material (Additional file 1: Figs. S2–S6; Table S2).

### Intracranial pressure

**Figures 1:**
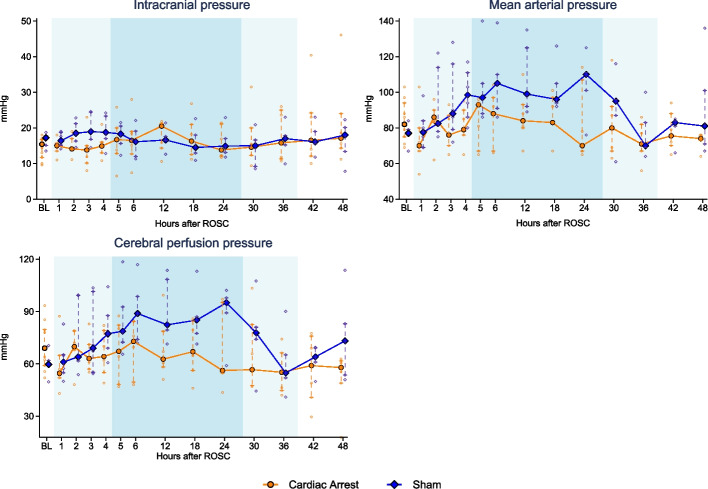
Intracranial, mean arterial, and cerebral perfusion pressure in the cardiac arrest and sham groups. Data are presented as median [25%; 75%] with overlaid scatter representing each animal. Lighter blue shading indicates induction/rewarming phase, respectively, while darker blue shading indicates maintenance phase of targeted temperature management. *BL* baseline before induction of myocardial infarction and cardiac arrest

ICP, CerPP, and MAP are presented in Fig. 1. No statistically significant differences were seen between groups at baseline for ICP (cardiac arrest: 15 mmHg [12; 17] vs sham: 17 [15; 18], *p* = 0.12) or CerPP (cardiac arrest: 69 mmHg [59; 80] vs sham: 60 [59; 62], *p* = 0.06). In one animal in the cardiac arrest group ICP increased up to 46 mmHg at 48 h after resuscitation, but on visual inspection, ICP otherwise developed similarly between groups and no difference was found between groups at the end of the protocol (cardiac arrest: 17 mmHg [14; 24] vs sham: 18 [13; 20], *p* = 0.44). Accordingly, the CerPP followed the development of the arterial pressure, with elevated levels during the maintenance phase of TTM, and no statistically significant difference was found between groups for CerPP 48 h after resuscitation (cardiac arrest: 60 mmHg [49; 62] vs sham: 73 [54; 83], *p* = 0.25).

### Cerebral oxygenation

**Fig. 2:**
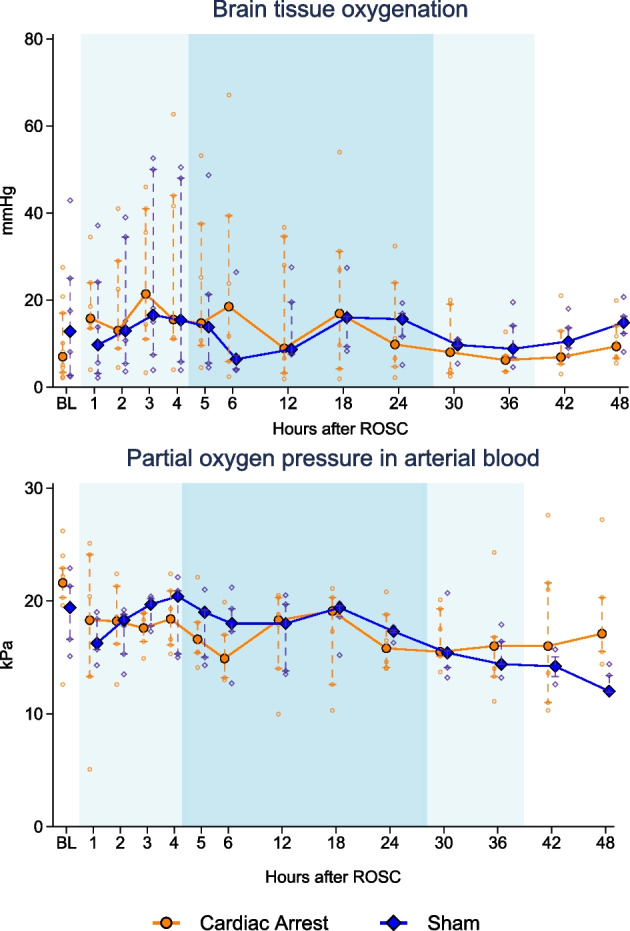
Brain oxygenation and partial pressure of O_2_ in arterial blood in the cardiac arrest and sham groups. Data are presented as median [25%;75%] with overlaid scatter representing each animal. Lighter blue shading indicates induction/rewarming phase, respectively, while darker blue shading indicates maintenance phase of targeted temperature management. *p < 0.01 between groups at 48 h. *BL* baseline before induction of myocardial infarction and cardiac arrest

PTiO_2_ and PaO_2_ are presented in Fig. 2. There was no statistically significant difference between groups at baseline for PTiO_2_ (cardiac arrest: 7 mmHg [3; 17] vs sham: 13 [3; 25], *p* = 0.50). By inspection of scatter plot, large between-animal variations existed in PTiO_2_ in both groups during the first 12 h of recording, but PTiO_2_ overall developed similarly between groups. The amplitudes of all PTiO_2_ measurements were above 400. No statistically significant difference was seen at 48 h (cardiac arrest: 9 mmHg [7; 14] vs sham: 15 [12; 16], *p* = 0.28). The Spearman correlation coefficient between PaO_2_ and PTiO_2_ was − 0.02 (95%CI − 0.16 to 0.13, *p* = 0.84, see Additional file 1: Fig. S6A).

### Microdialysis

**Fig. 3:**
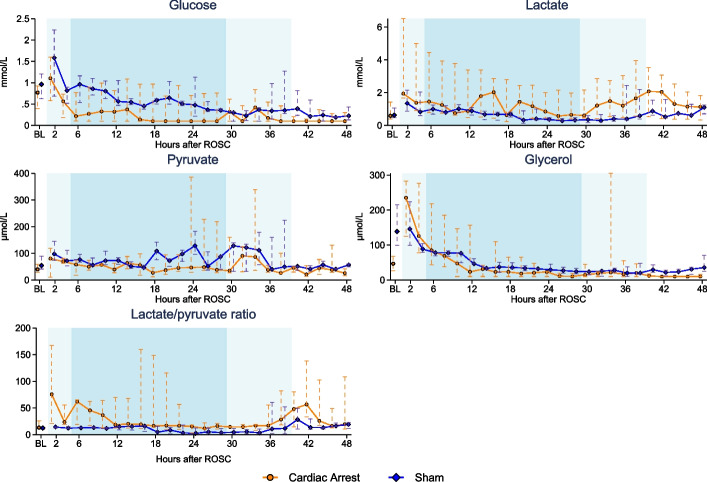
Cerebral microdialysis analytes in the cardiac arrest and sham groups. Data are presented as median [25%; 75%]. Lighter blue shading indicates induction or rewarming phase, while darker blue shading indicates maintenance phase of targeted temperature management

Cerebral microdialysis data are presented in Fig. 3. Median glucose levels rose from baseline in both groups during induction of TTM, however, to a higher level in the sham group compared with the cardiac arrest group. Glucose levels remained lower in the cardiac arrest group throughout the maintenance phase of TTM. Glucose levels in the sham group slowly declined during induction and maintenance phase and from the rewarming phase until end-of-protocol glucose levels were similar between groups. Median lactate levels were slightly higher in the cardiac arrest group during most of the protocol after cardiac arrest. Of interest, there was an increase in median lactate levels during the rewarming phase of TTM in the cardiac arrest group, which was not observed in the sham group. Pyruvate levels were overall similar between the two groups up until the rewarming phase. An increase in pyruvate was observed in both groups at the beginning of rewarming. The lactate/pyruvate (L/P) ratio was higher in the cardiac arrest group throughout the first 10 h after resuscitation and during the last part of the rewarming phase. Glycerol levels were higher at baseline in the sham group compared with the cardiac arrest group. Despite this, glycerol levels increased more in the cardiac arrest group two hours after resuscitation. Both groups had similarly declining glycerol levels throughout the remainder of the protocol. Individual scatter plots for lactate, pyruvate and their ratio are presented for the cardiac arrest group in Additional file 1: Fig. S7.

### Blood markers of cerebral injury

**Fig. 4:**
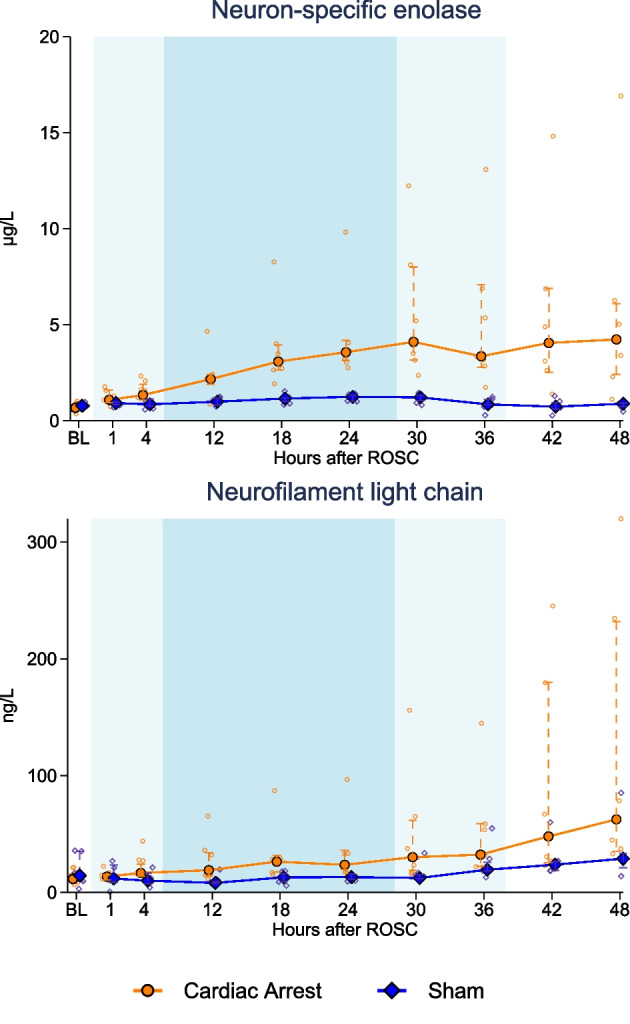
Level of neuron-specific enolase and neurofilament light chain over time in the cardiac arrest and sham groups. Data are presented as median [25%; 75%] with overlaid scatter plots representing each animal. Lighter blue shading indicates induction or rewarming phase, while darker blue shading indicates maintenance phase of targeted temperature management

NSE and NfL levels are depicted in Fig. 4. The repeated measurements analysis showed that the NSE increased significantly over time (*p* < 0.0001), and at 48 h after resuscitation the relative between-group difference was 5.19 (95%CI 3.08–8.74, *p* < 0.001) with values of 4.2 µg/L [2.4; 6.1] vs 0.9 [0.7; 0.9]. NfL likewise increased over time in the cardiac arrest group (*p* = 0.0001) reaching a larger relative values of 2.68 (95%CI 1.22–5.90, *p* = 0.02) at 48 h after resuscitation with values of 63 ng/L [35; 232] vs 29 [21; 34]. The Spearman correlation analysis of NSE and NfL in the cardiac arrest group revealed a coefficient of 0.74 (95%CI 0.62–0.83, *p* < 0.0001, see Additional file 1: Fig. S6B).

### MRI and MR spectroscopy

**Fig. 5:**
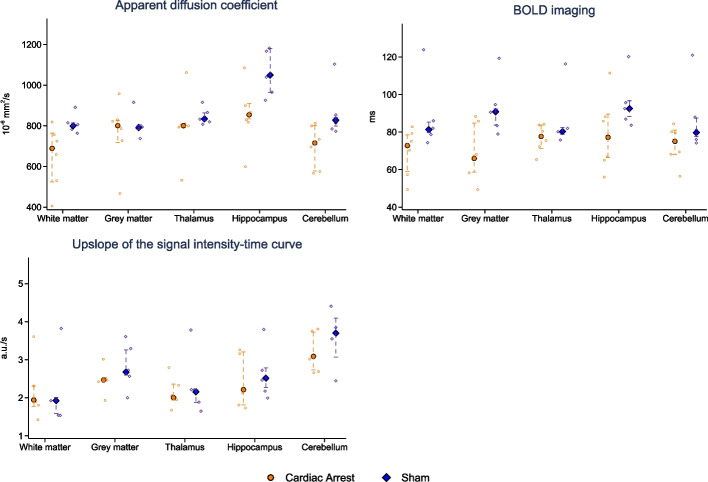
Apparent diffusion coefficient values, blood oxygenation level dependent (BOLD) imaging, and upslope of signal intensity curve of perfusion-weighted imaging in five different anatomical locations in the brain. Data are presented as median [25–75%] with overlaid scatter plots representing each animal. *Ms *milliseconds, *a.u.* arbitrary unit

**Fig. 6:**
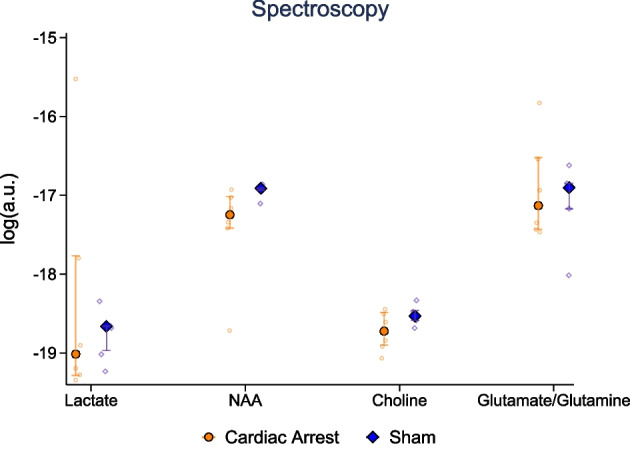
MR spectroscopy with concentrations (in arbitrary units) for four metabolites from a whole brain voxel. *Y*-axis is presented on log 10 scale. Data are presented as median [25–75%] with overlaid scatter plots representing each animal. *NAA* *N*-acetylaspartate, *a.u. *arbitrary units

ADC, BOLD imaging, and upslope of the signal intensity–time curve results are presented in Fig. 5. In the cardiac arrest group, significantly lower ADC values were seen in white matter frontal cortex (689 × 10^–6^ mm^2^/s [524; 765] vs 800 [799; 815], *p* = 0.04) and hippocampus (854 × 10^–6^ mm^2^/s [834; 910] vs 1049 [965; 1180] *p* = 0.03), but no statistical significant difference was observed in grey matter (801 × 10^–6^ mm^2^/s [717; 826] vs 791 [781; 801], *p* = 0.93), thalamus (801 × 10^–6^ mm^2^/s [796; 805] vs 834 [824; 863], *p* = 0.18), or cerebellum (715 × 10^–6^ mm^2^/s [579; 802] vs 828 [798; 841], *p* = 0.05). The BOLD sequence showed that relaxation time, as a measure of deoxygenated blood levels, in the cardiac arrest group was statistical significantly lower in grey matter cortex (66 ms [59; 85] vs 91 [84; 92], *p* = 0.03). Lower values without statistical significance was seen in the cardiac arrest group in white matter cortex (73 ms [59; 79] vs 81 [80; 85], *p* = 0.52), thalamus (78 ms [71; 84] vs 80 [79;83], *p* = 0.54), hippocampus (77 ms [66; 90] vs 93 [88; 97], *p* = 0.18), and cerebellum (75 ms [68; 81] vs 80 [78; 87], *p* = 0.33). For the upslope of the signal intensity–time curve as a measure of perfusion, no differences were seen at any of the anatomical locations. MR spectroscopy metabolite levels are presented in Fig. 6. NAA was lower in the cardiac arrest group (− 17.2 log [− 17.4; − 17.0] vs − 16.9 [− 16.9; − 16.9], *p* = 0.03). No between-group difference (cardiac arrest vs sham) was observed for lactate (− 19.0 log [− 19.3; − 17.8] vs − 18.7 [− 19.0; − 18.7], *p* = 0.79), choline (− 18.7 log [− 18.9; − 18.5] vs − 18.5 [− 18.6; − 18.5], *p* = 0.14), or glutamate/glutamine (− 17.1 log [− 17.4; − 16.5] vs − 16.9 [− 17.2; − 16.8], *p* = 0.85) peaks in the spectroscopy data.

## Discussion

Overall, our pig model of cardiac arrest demonstrated neuronal injury by elevated blood markers. Furthermore, signs of post-ischaemic cytotoxic oedema were shown on MRI ADC maps and decreased neuronal viability by NAA on proton-spectroscopy. Microdialysis data showed impaired cerebral metabolism in the cardiac arrest group, which was most pronounced during the first 10 h after resuscitation.

### Intracranial pressure and cerebral perfusion

The CerPP was seemingly higher during the maintenance phase of TTM in the sham group. The interpretation of this difference is difficult because the intensive care protocol used, only ensured a MAP > 65 mmHg using vasopressor treatment. Hence, the difference in CerPP is caused by a higher MAP in the sham group. Interestingly, the ICP remained at similar levels in the cardiac arrest and sham groups during the intensive care period, disregarding the one cardiac arrest animal that developed a very high ICP. The ICP during cardiac arrest, resuscitation, and the immediate ROSC period is well described in animal models. ICP rises upon the cessation of cardiac function, and then gradually falls or stays elevated during resuscitation depending on the quality and mode of CPR [10, 14–16]. The increase during cardiac arrest and resuscitation is due to decreased venous return, as proven by decreased ICP values during CPR which incorporates active measures to decrease intrathoracic pressure [22]. Looking beyond the immediate post-resuscitation phase, case series of hypoxic–ischaemic brain injury patients have reported differentiation of poor vs. good neurological prognosis by looking at ICP during the first 5–6 days after ROSC [6, 9]. Other studies have shown a generally low prevalence of intracranial hypertension after cardiac arrest, although with a trend towards higher ICP values in non-survivors [23–25]. This picture of an overall low prevalence of intracranial hypertension is reproduced in our study. In some cases, high ICP levels indicate a higher degree of cerebral injury when also considering the two animals from the AMI-Cont group with intracranial hypertension and high NSE values. We did not see any major deviations in ICP during cooling or rewarming in contrast with previous investigations in pigs and humans [7, 26]. Although there is an apparent association between very high ICP values and increased cerebral injury, results from recent studies in both pigs and humans do agree that using ICP and blood pressure correlation analysis for indirect measurements of intracranial compliance or cerebral autoregulation shows stronger associations than ICP alone [9, 12, 23]. Unfortunately, due to the temporal granularity of our data, we were not able to conduct such analysis.

### Brain oxygenation

The lower limit for brain normoxia is considered 20 mmHg and the PTiO_2_ measurements in our study were lower in both groups [8]. Of importance, PTiO_2_ increased with FiO_2_ elevations and fell accordingly during cardiac arrest indicating the probe was responsive to changes in oxygen levels [2]. Despite the low values, we can derive that the PTiO_2_ levels seemingly did not vary much between groups over the course of the intensive care period. A normal L/P ratio and normal biomarkers was seen throughout the protocol for sham pigs, which indicates the absence of brain hypoxia. Furthermore, the amplitude of the PTiO_2_ signal was above the level of the manufactures recommendation why we do not consider dampening of the signal as a factor. A large variance was seen during the first 6 h after resuscitation, due to probe stabilization, altered brain perfusion/metabolism in cardiac arrest animals, PaO_2_ titration determined by the protocol, and effect of TTM induction. The PTiO_2_ levels in this study were not associated with PaO_2_, which is in line with a previous investigation showing that PaO_2_ only describes a small portion of PTiO_2_ variance. [13]

### Cerebral metabolism

To our knowledge, this is the first study of long-term cerebral metabolism after resuscitation with a high temporal solution in a controlled experimental setting. Markers of cerebral metabolism during cardiac arrest and resuscitation has been described with an immediate rise in lactate, glycerol, and L/P ratio, as well as a drop in glucose [10, 11, 27]. In our study, we saw a high peak in L/P ratio in the cardiac arrest group at 2 h after resuscitation. The microdialysis vials in our study were changed 5 min after successful resuscitation, so this early peak to some extent represents the no/low flow periods. Beyond 2 h, two different patterns emerged in the cardiac arrest group: (1) four animals had normalization of L/P ratio, and (2) three animals experienced secondary/tertiary surges in L/P ratio (see Additional file 1: Fig. S7). The exact mechanisms generating the secondary L/P ratio elevation could be manifold, e.g., microcirculatory dysfunction, neurovascular uncoupling, mitochondrial dysfunction, etc. In our data, secondary surges in L/P ratio were mainly accompanied by low pyruvate levels, indicating ischaemic/hypoxic conditions, and not mitochondrial dysfunction. Previous studies of cerebral metabolism have shown similar patterns as we observed: Yuan et al. have in two studies shown elevations in L/P ratio of around 50–100 between 6–16 h after resuscitation indicating secondary surges [28, 29], whereas Skåre et al. on the contrary showed early peak values of L/P, with a rapid near-normalization of L/P ratio during the first hour after resuscitation in a 10-min no-flow pig model [12]. The studies by Yuan et al. had low temporal granularity and the development over time is therefore difficult to interpret. The model by Skåre et al. utilized resuscitative efforts with high-flow cardiopulmonary bypass ensuring near-optimal macrocirculatory conditions prior to ROSC, which could have led to a faster recovery of cerebral metabolism. Further studies into the mechanisms of dysregulated cerebral metabolism, perhaps with focus on linking disturbances to vascular dysfunction, after successful resuscitation, are needed.

In a case series of 10 post-cardiac arrest patients, Hifumi et al. demonstrated increasing L/P ratios over the first four days after ROSC. Furthermore, they showed a correlation between increasing ICP levels and L/P ratio in a subgroup of patients with poor neurological outcome [25]. On this basis, they proposed a theory of brain oedema and likely ICP elevations as the reason for the cerebral metabolic disturbances. Our data showed downward trends in PTiO_2_ and cerebral metabolic disturbances prior to ICP elevations in pigs with intracranial hypertension (including the two animals from the AMI-Cont group, see Additional file 1: Fig. S2). This indicates reperfusion injury and/or secondary hypoxia as the instigating cause of cerebral oedema and ICP elevation and not vice versa.

### Blood markers

NSE is, as of now, the only recommended biomarker of cerebral injury to be used in prognostication of cardiac arrest survivors [30]. In recent retrospective observational studies NfL has been shown to be predictive of poor neurologic outcome as early as on hospital admission with peak values occurring around 48 h after admission [31–33]. To our knowledge this is the first study to investigate NfL in a pre-clinical model of cardiac arrest. Although NfL rose with the same kinetics as NSE the relative difference was smaller when compared with sham pigs.

### MRI

For assessment of cerebral oedema by ADC maps in human studies, a threshold value in a proportion of the brain is often measured and growing evidence is supporting a threshold of < 650 × 10^–6^ mm^2^/s in > 10% of the brain [34]. For this study, although the ICP/PTiO_2_ probes in the pigs were removed before the scan, an MRI-sensible artefact was still present in the location of the catheter, which precluded a volume-ratio measurement. Therefore, we took the approach of locating ROIs in specific anatomical locations. Our findings with lower ADC values in the cardiac arrest group in white matter cortex and hippocampus (and lower in cerebellum but not significant) indicate that cerebral oedema formation did occur as a result of the ischaemia–reperfusion injury elicited by cardiac arrest. Diffusion-weighted imaging with ADC maps has the sensitivity to detect cytotoxic oedema [35]. In the cardiac arrest pigs with normal range ICP at the end of the protocol lower ADC values may therefore be indicative of cytotoxic oedema.

The cause of the relatively large ADC values in the hippocampi in the sham group is unknown. To exclude the possibility of cerebrospinal fluid from the lateral or third ventricles (which would otherwise increase ADC values) the hippocampal ROIs were minimized in a secondary analysis, but this yielded no change in ADC values.

The lower relaxation time in grey matter cortex from BOLD imaging, demonstrates a higher relative concentration of deoxyhemoglobin. Coinciding this finding with the normal perfusion measures indicates a larger metabolic demand than supply. When inspecting the relaxations times from all five regions there is a trend towards lower values in the cardiac arrest group in all regions. These results should be interpreted with caution due to a low number of animals undergoing MRI, but they could indicate a persisting mismatch in the demand and supply of oxygen in the vulnerable brain after cardiac arrest. Furthermore, although not significant, median PTiO_2_ levels did trend towards lower values in the cardiac arrest group. Reports in humans have shown secondary hypoxia as the cause of de novo neuronal damage. [8]

### Limitations

Although the pig has many physiological and anatomical similarities with humans, establishing a direct link between the results from this study to the clinical setting is not possible. Furthermore, there were a relatively low number of animals in each group. Treatment of high ICP or low PTiO_2_ levels was not included in the study protocol, as either parameter could have affected other outcome measurements in this study (e.g., microdialysis and MRI). The microdialysis data only represent one area of the brain, and although the ischaemic insult after cardiac arrest is global, injury progression is different between anatomical locations.

## Conclusion

We have developed a clinically relevant cardiac arrest pig model that displays cerebral injury as marked by NSE and NfL elevations, signs of cerebral oedema, and reduced neuron viability. Overall, the burden of elevated ICP was low in the cardiac arrest group. A subset of pigs undergoing cardiac arrest had persisting metabolic disturbances after successful resuscitation.

## Supplementary Information

**Additional file 1: Table S1**. Post-cardiac arrest intensive care protocol. Table S2 Haemoglobin, blood lactate and pH. **Figure S1** Regions of interest displayed on T1 weighted MRI scan. **Figure S2** Pressure and oxygen levels for both cardiac arrest groups and sham controls. **Figure S3** Microdialysis data for both cardiac arrest groups and sham controls. **Figure S4** MRI and MR spectroscopy for both cardiac arrest groups and sham controls. **Figure S5** Neuronal injury markers for both cardiac arrest groups and sham controls. **Figure S6** Scatter plot of blood/brain oxygenation and NSE/NfL levels for each animal. **Figure S7** Scatter plots of lactate, pyruvate, and their ratio from animals in AMI-Int cardiac arrest group.

## Data Availability

The datasets used and/or analysed during the current study are available from the corresponding author on reasonable request.

## Abbreviations

ADC: Apparent diffusion coefficient
AMI-Cont: Cardiac arrest group with continuously myocardial infarction
AMI-Int: Cardiac arrest group with reperfusion of coronary artery prior to cardiac arrest and re-occlusion upon ROSC
BOLD: Blood oxygen level dependent
CerPP: Cerebral perfusion pressure
FiO_2_: Fraction of inspired oxygen
ICP: Intracranial pressure
L/P: Lactate/pyruvate
MAP: Mean arterial blood pressure
MRI: Magnetic resonance imaging
NAA: *N*-Acetylaspartate
NSE: Neuron specific enolase
NfL: Neurofilament light chain
PaCO_2_: Partial pressure of CO_2_ in arterial blood
PaO_2_: Partial pressure of O_2_ in arterial blood
PTiO_2_: Brain tissue oxygenation
ROSC: Return of spontaneous circulation
SvO_2_: Mixed venous blood oxygen saturation
TTM: Targeted temperature management
