# Supplementary material for: Cerebral monitoring in a pig model of cardiac arrest with 48 h of intensive care
Source: Intensive Care Med Exp. 2022 Oct 26;10:45. doi: 10.1186/s40635-022-00475-2 (PMC9596181; doi:10.1186/s40635-022-00475-2)
Supplement: Supplementary file 1 — Additional file 1: Table S1. Post-cardiac arrest intensive care protocol. Table S2 Haemoglobin, blood lactate and pH. Figure S1 Regions of interest displayed on T1 weighted MRI scan. Figure S2 Pressure and oxygen levels for both cardiac arrest groups and sham controls. Figure S3 Microdialysis data for both cardiac arrest groups and sham controls. Figure S4 MRI and MR spectroscopy for both cardiac arrest groups and sham controls. Figure S5 Neuronal injury markers for both cardiac arrest groups and sham controls. Figure S6 Scatter plot of blood/brain oxygenation and NSE/NfL levels for each animal. Figure S7 Scatter plots of lactate, pyruvate, and their ratio from animals in AMI-Int cardiac arrest group. [file 40635_2022_475_MOESM1_ESM.docx]

**Cerebral monitoring in a pig model of cardiac arrest with 48 hours of intensive care**

**Supplemental material**

**Table of content**

[Magnetic resonance imaging supplemental description 3](#_Toc113457563)

[Table S1. Post-cardiac arrest intensive care protocol 4](#_Toc113457564)

[Table S2 – Hemoglobin, blood lactate and pH 7](#_Toc113457565)

[Figure S1 – Regions of interest displayed on T1 weighted MRI scan 8](#_Toc113457566)

[Figure S2 – Pressure and oxygen levels for both cardiac arrest groups and sham controls 9](#_Toc113457567)

[Figure S3 – Microdialysis data for both cardiac arrest groups and sham controls 10](#_Toc113457568)

[Figure S4 – MRI and MR spectroscopy for both cardiac arrest groups and sham controls 11](#_Toc113457569)

[Figure S5 – Neuronal injury markers for both cardiac arrest groups and sham controls 12](#_Toc113457570)

[Figure S6 – Scatter plot of blood/brain oxygenation and NSE/NfL levels for each animal 13](#_Toc113457571)

[Figure S7 – Scatter plots of lactate, pyruvate, and their ratio from animals in AMI-Int cardiac arrest group. 14](#_Toc113457572)

# Magnetic resonance imaging supplemental description

*Magnetic resonance imaging*

A standard Philips head RF coil was used for data acquisition. After localizer imaging, 3D T1-weighted and T2-weighted sequences were applied followed by imaging sequences for measuring water diffusion, blood oxygenation, proton spectroscopy, and blood perfusion.

The T1-weighted sequence was a 3D gradient echo sequence with an inversion pre-pulse. Field-of-view (FOV) was 250×250×170 mm, acquisition pixel size was 1.0×1.0×1.0 mm, TR/TE/TI were 9.3/4.6/1050 ms and flip angle was 8°. The T2-weighted sequence was a 3D fast spin echo sequence with varying refocus flip angle. FOV was 250x250x178 mm, acquisition pixel size was 1.1x1.1x1.1 mm, and TR/TE were 2800/244 ms.

For assessing tissue oxygenation was indirectly measured by a blood oxygenation level dependent (BOLD) sequence. Sixteen axial slices with 12 echotime between 5 and 71 ms were acquired. FOV was 220×220 mm, slice thickness was 5 mm, and pixel size was 1.5×1.5 mm. TR was 1158 ms and flip angle was 25°.

The diffusion-weighted sequence was based on a fast spin-echo sequence with diffusion-weighting b-values of 0 and 800 s/mm^2^. Twenty-two axial slices with slice thickness of 5 mm, FOV of 230×230 mm, and pixel size of 1.2×1.2 mm were sampled. TR/TE were 4184/114 ms.

For measuring blood perfusion, gadolinium based contrast agent was injected during continuous scanning with a single-shot gradient-echo EPI sequence. Six axial slices with slice thickness of 8 mm, FOV of 200x200 mm, and pixel size of 1.5x1.5 mm were sampled. TR/TE was 500/40 ms, and 240 dynamics were acquired.

The spectroscopy sequence was a single-shot PRESS sequence with voxel size of 25×25×50 mm. TR/TE were 2000/135 ms, Spectral bandwidth was 1000 Hz, 512 points were sampled, and number of averages was 192.

# Table S1. Post-cardiac arrest intensive care protocol

Treatment goals;

- PaCO_2_ 4,6-6.0 kPa
- SaO_2_ 94-98 %
- MAP >65 mmHg
- Cardiac output >2.0 L/min
- SvO_2_ >50 %
- Blood (B)-glucose 4-10 mmol/L
- B-[K^+^] 3.0-5.5 mmol/L
- Urine output >1 ml/kg/hr

| **CNS** | |
| --- | --- |
| Treatment goal | Interventions |
| - Avoid seizures | Continued sedation with propofol and remifentanil as per anesthesia protocol (see *Supplemental methods description*).  If convulsions;   1. Propofol bolus 1 mg/kg 2. Midazolam 0.3 mg/kg |
| **RESPIRATION** | |
| Treatment goal | Interventions |
| - PaCO_2_ = 4.7-6.0 kPa - SaO_2_ = 94-98 % | Initial ventilator settings;   - Pressure controlled with volume guarantee (PCV-VG) - Tidal volume = 8 ml/kg - Respiratory rate = 10-35 per min (adjusted to PaCO_2_) - FiO_2_ = 0.30 - PEEP = 5 cm H_2_O   A sustained (>10 min) decrease in blood oxygen levels (SaO_2_) was intervened by first suctioning the tracheal tube. If insufficient, increase FiO_2_ (10 %) and PEEP (2 cm H_2_O), respectively, starting with the value furthest from initial values; if equidistant starting with PEEP. A sustained increase in oxygen levels was intervened on conversely. Lowest PEEP level at 5 cm H_2_O.   | **FiO_2_** (%) | 0.4 | 0.5 | 0.6 | 0.7 | 0.8 | 0.9 | 1.0 | | --- | --- | --- | --- | --- | --- | --- | --- | | **PEEP** (cm H_2_O) | 5 | 7 | 9 | 11 | 13 | 15 | 16-24 | |
| **CARDIOVASCULAR** | |
| Treatment goal | Interventions |
| - MAP > 65 mmHg - SvO_2_ > 50 % - Cardiac output (CO) > 2 L/min - Cardioversion to sinus rhythm | Immediately upon ROSC, a 10 ml/kg unheparinized fluid bolus was given.  Hemodynamic  **↓***CO/SvO_2_*   1. Fluid bolus (4 ml/kg)    1. Increase in SvO_2_/CO 🡪 repeat    2. No effect and/or target not reached 🡪 dobutamine    3. Maximum fluid = 1 L/24 hours post ROSC 2. Dobutamine; 0.1-15.0 µg/kg/min    1. Increased dosage until target SvO_2_/CO reached   *N.B. if SvO_2_ drops below 50 % but CO>2.5 L/min give fluid bolus to effect but do not start dobutamine.*  **↓***MAP*   1. Noradrenaline; 0.01-1.00 µg/kg/min    1. Increased dosage until target MAP reached 2. Terlipressin    1. Start if vasoplegic despite max noradrenaline and normal cardiac output/SvO_2_.    2. Bolus treatment until effect (bolus size = 0.2 mg, max 1 mg) 3. Within initial 60 min of ROSC if noradrenaline is insufficient, repeat adrenaline boluses (0.01-0.1 mg) titrated to reach target MAP. 4. If MAP <65 mmHg despite max dosage of noradrenaline and terlipressin, start adrenaline infusion 0.01-1.00 µg/kg/min.   Arrhythmia   1. Major cardiovascular instability;    1. Ventricular fibrillation/pulseless VT; 3 stacked shocks, 360 J with rhythm checks in between, if no effect start CPR.    2. Asystole/PEA; start CPR as per guidelines, i.e. cycles of 2 min of chest compressions and mechanical ventilation with rhythm check in between and adrenaline and/or amiodarone as recommended. 2. Cardiovascular stable;    1. Persistent ventricular tachycardia (VT); 3 stacked synchronized shocks 360 J with rhythm checks in between, if insufficient or if intermittent VT 300 mg amiodarone i.v. repeated until cardioversion (max 1,200 mg/24 hr). If max dose reached give 1 mg/kg lidocaine and if necessary start cont. infusion 1 mg/min    2. Supraventricular tachycardia with hemodynamic instability and/or HR>120; 3 synchronized shocks 360 J, if insufficient 300 mg amiodarone i.v. repeated every hour until cardioversion   Amiodarone is administered over 10 min to avoid hypotension. Maximum amiodarone dose = 1200 mg/24 hr. |
| **GASTROINTESTINAL** | |
| Intervention | |
| 10 ml/hr enteral nutrition via feeding tube started post-ROSC (equivalent to 3 drops per minute.) | |
| **RENAL** | |
| Treatment goal | Interventions |
| - Urine output > 1 ml/kg/hr - B-[K^+^] 3.0-5.5 mmol/L | - Basal i.v. fluid replacement with Ringer’s lactate 2 ml/kg/hr. If [Na^+^] falls below 137 mmol/L both basal and bolus fluid treatment is exchanged with isotonic NaCl. - If hypokalemia KCl is mixed with the basal fluid replacement. Total supplement given calculated by [3.5 mmol/L]-[actual K^+^]*[body weight] - If hyperkalemia;   - 10 mg furosemide to effect, if insufficient 🡪   - 10 IU rapid acting insulin mixed in 50 ml 50 % glucose administered over 5 min.   - If hyperkalemia and arrhythmia, 5 mmol Ca^2+^. |
| **INFECTION** | |
| Intervention | |
| 750 mg Cefuroxim every 8 hours post ROSC. | |
| **COAGULATION** | |
| Treatment goal | Intervention |
| Prevent thrombo-embolic events | Unfractionated heparin 18 IU/kg/hr (in addition 10,000 IU in total administered during cardiac catheterization). |
| **ENDOCRINOLOGY** | |
| Treatment goal | Intervention |
| - B-[glucose] 4-10 mmol/L | | Blood glucose (mmol/L) | Insulin dose (rapid acting) | | --- | --- | | 10-12 | 4 IE | | 12-16 | 6 IE | | 16-20 | 8 IE | | >20 | 10 IE | | Dose repeated after minimum 1 hr  NB; watch for hypoglycemia during rewarming | |   If blood glucose < 4 mmol/L, a bolus of 20 ml 50 % glucose solution is administered over 5 min with blood glucose control after 15 min. |

# Table S2 – Hemoglobin, blood lactate and pH

|  | **GROUP** | **Baseline** | **1 hour** | **4 hours** | **12 hours** | **18 hours** | **24 hours** | **30 hours** | **36 hours** | **42 hours** | **48 hours** |
| --- | --- | --- | --- | --- | --- | --- | --- | --- | --- | --- | --- |
| **TTM phases** |  |  | Start TTM | Target temp. | Maintenance phase | | | Rewarming | | Active normothermia | |
| **Hb** (mmol/L) | **AMI-Cont** | 5.6 [5.5:5.8] | 6.8 [6.2:7.4] | 7.0 [6.6:7.3] | 6.3 [6.2:6.6] | 6.2 [6.0:6.7] | 5.3 [5.1:6.8] | 6.0 [5.5:6.4] | 5.4 [4.2:6.0] | 5.4 [4.1:5.9] | 4.8 [3.6:5.2] |
|  | **AMI-Int** | 5.8 [4.9:6.0] | 6.3 [6.0:7.6] | 6.2 [5.7:7.5] | 5.7 [5.2:6.6] | 6.2 [5.6:6.9] | 6.2 [5.9:7.3] | 6.8 [5.9:7.1] | 5.7 [5.3:6.3] | 5.4 [5.2:5.4] | 4.8 [4.4:5.3] |
|  | **Sham** | 5.4 [5.1:5.9] | 5.5 [5.2:5.7] | 6.2 [6.0:6.3] | 6.7 [6.2:6.8] | 6.8 [6.3:7.3] | 6.1 [6.1:7.1] | 6.0 [6.0:6.7] | 5.7 [5.6:6.3] | 5.5 [5.3:5.6] | 5.5 [4.9:5.8] |
| **B-Lactate** (mmol/L) | **AMI-Cont** | 0.6 [0.5:1.0] | 7.2 [5.4:11.9] | 4.8 [2.5:11.8] | 0.9 [0.6:4.4] | 4.2 [0.6:6.5] | 0.9 [0.8:1.1] | 1.7 [0.6:7.4] | 1.3 [0.4:3.7] | 2.6 [0.5:2.8] | 1.1 [0.6:2.4] |
|  | **AMI-Int** | 0.8 [0.7:0.9] | 5.8 [5.2:11.0] | 2.0 [1.0:6.1] | 0.9 [0.6:1.3] | 1.7 [0.6:7.5] | 1.2 [0.8:2.8] | 1.0 [0.6:3.4] | 1.0 [0.6:5.0] | 1.2 [0.4:2.5] | 0.8 [0.4:1.2] |
|  | **Sham** | 0.6 [0.5:0.6] | 0.6 [0.5:0.6] | 0.4 [0.4:0.5] | 0.5 [0.4:0.5] | 0.4 [0.3:0.7] | 0.4 [0.4:0.4] | 0.4 [0.3:0.5] | 0.4 [0.4:0.5] | 0.4 [0.4:0.5] | 0.4 [0.3:0.4] |
| **pH** | **AMI-Cont** | 7.47 [7.46:7.52] | 7.30 [7.05:7.36] | 7.32 [7.13:7.34] | 7.39 [7.34:7.52] | 7.36 [7.33:7.55] | 7.46 [7.42:7.53] | 7.46 [7.24:7.47] | 7.39 [7.36:7.43] | 7.41 [7.40:7.42] | 7.41 [7.35:7.50] |
|  | **AMI-Int** | 7.49 [7.48:7.51] | 7.34 [7.18:7.37] | 7.40 [7.30:7.46] | 7.47 [7.41:7.52] | 7.45 [7.34:7.57] | 7.47 [7.40:7.50] | 7.44 [7.42:7.51] | 7.42 [7.24:7.44] | 7.45 [7.40:7.45] | 7.48 [7.43:7.50] |
|  | **Sham** | 7.47 [7.45:7.52] | 7.49 [7.48:7.51] | 7.44 [7.43:7.49] | 7.52 [7.45:7.53] | 7.50 [7.49:7.52] | 7.51 [7.46:7.52] | 7.51 [7.47:7.51] | 7.50 [7.44:7.52] | 7.53 [7.48:7.54] | 7.48 [7.48:7.48] |
| Data presented as median[25%;75%]. Time points after baseline represents hours since return of spontaneous circulation/sham intervention. TTM: target temperature management, Hb: hemoglobin, B: blood. | | | | | | | | | | | |

# Figure S1 – Regions of interest displayed on T1 weighted MRI scan

Representative images of regions of interest from T1 weighted MRI scans. A) grey and white amatter cortex, B) thalamus, C) hippocampus, D) Cerebellum.


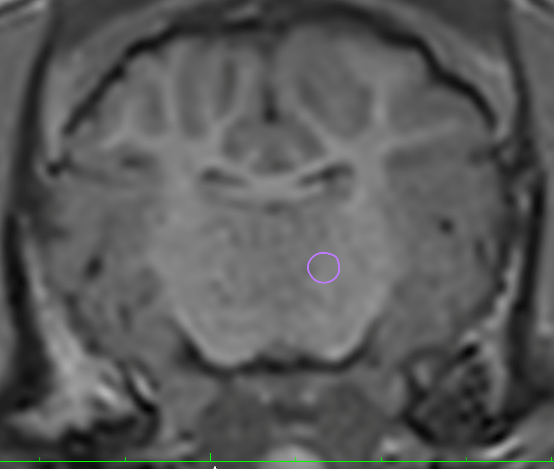

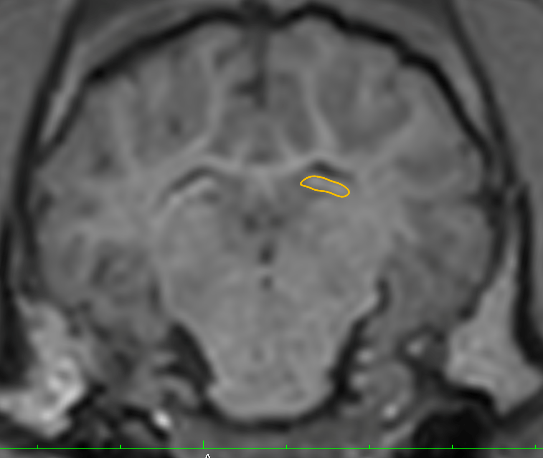

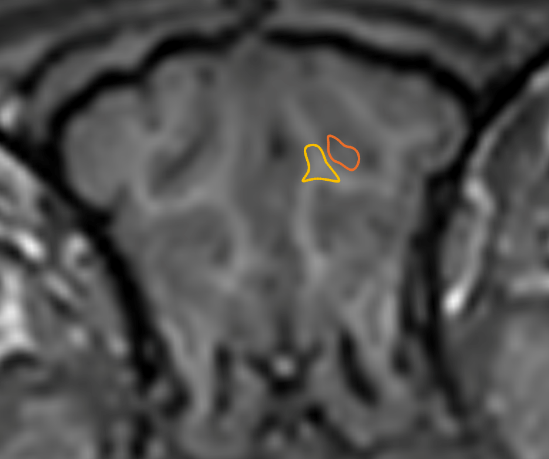

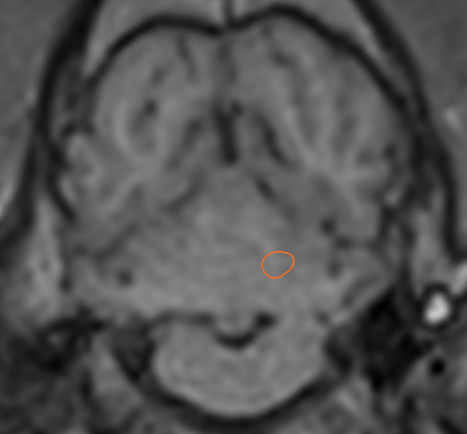


**A**

**C**

**B**

**D**

# Figure S2 – Pressure and oxygen levels for both cardiac arrest groups and sham controls

Data presented as median [25%;75] with overlaid scatter plots representing each animal. Lighter blue shading indicates induction/rewarming phase respectfully, while darker blue shading indicates maintenance phase of targeted temperature management. AMI Int: cardiac arrest group with moderate post-cardiac arrest phenotype (Cardiac arrest group in main manuscript), AMI Cont: cardiac arrest group with severe post-cardiac arrest phenotype.

# Figure S3 – Microdialysis data for both cardiac arrest groups and sham controls

Data presented as median [25%;75] with overlaid scatter plots representing each animal. Lighter blue shading indicates induction/rewarming phase respectfully, while darker blue shading indicates maintenance phase of targeted temperature management. AMI Int: cardiac arrest group with moderate post-cardiac arrest phenotype (Cardiac arrest group in main manuscript), AMI Cont: cardiac arrest group with severe post-cardiac arrest phenotype.

# Figure S4 – MRI and MR spectroscopy for both cardiac arrest groups and sham controls

Data presented as scatter plots (representing each animal) because only two animals in the AMI Cont group survived until MRI scan which precluded further descriptive statistics. AMI Int: cardiac arrest group with moderate post-cardiac arrest phenotype (Cardiac arrest group in main manuscript), AMI Cont: cardiac arrest group with severe post-cardiac arrest phenotype. Ms = milliseconds, a.u. = arbitrary unit

# Figure S5 – Neuronal injury markers for both cardiac arrest groups and sham controls

Data presented as median [25%;75] with overlaid scatter plots representing each animal. Lighter blue shading indicates induction/rewarming phase respectfully, while darker blue shading indicates maintenance phase of targeted temperature management. AMI Int: cardiac arrest group with moderate post-cardiac arrest phenotype (Cardiac arrest group in main manuscript), AMI Cont: cardiac arrest group with severe post-cardiac arrest phenotype.

# Figure S6 – Scatter plot of blood/brain oxygenation and NSE/NfL levels for each animal

Scatter plot of A) PaO_2_ and PTiO_2_ and B) NSE and NfL measurements. Both graphs represent data from all time points from both groups. Spearman correlation coefficients is based on data from both groups for PaO_2_/PtiO_2_ and only cardiac arrest group (AMI-Int) for NfL/NSE. PaO_2_ = partial oxygen pressure in arterial blood, PTiO_2_= brain tissue oxygenation, NSE = neuron specific enolase, NfL = neurofilament light chain

#
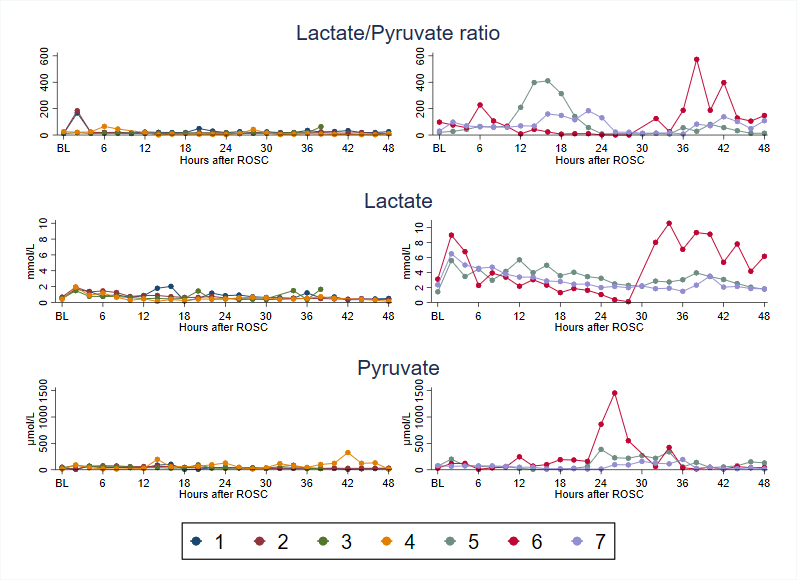
Figure S7 – Scatter plots of lactate, pyruvate, and their ratio from animals in AMI-Int cardiac arrest group.

Left column represent animals with normalization of lactate/pyruvate ratio beyond two hours after ROSC. Right column represents animals with secondary surges in lactate/pyruvate ratio. The different colors represent each animal.
